# Hemophagocytic Lymphohistocytosis in the Chinese Han Population May Be Associated with an STXBP2 Gene Polymorphism

**DOI:** 10.1371/journal.pone.0159454

**Published:** 2016-08-11

**Authors:** Li Yang, Yang Tang, Fang’Xi Xiao, Jie Xiong, Ke’Feng Shen, Ya’Nan Liu, Wei Zhang, Li’Chang Zheng, Jian’Feng Zhou, Min Xiao

**Affiliations:** 1 Department of Hematology, Tongji Hospital affiliated with Tongji Medical College, Huazhong University of Science and Technology, Hankou District, Wuhan, Hubei Province, China; 2 Department of Oncology, Tongji Hospital affiliated with Tongji Medical College, Huazhong University of Science and Technology, Hankou District, Wuhan, Hubei Province, China; 3 Department of Endocrinology, No. 1 Hospital, Qiaokou District, Wuhan, Hubei Province, China; National Cancer Institute, UNITED STATES

## Abstract

**Study Purpose:**

Hemophagocytic lymphohistiocytosis (HLH) is a life-threatening disease of severe hyperinflammation caused by uncontrolled proliferation of activated lymphocytes and macrophages. In this study, we aimed to explore the genetic factors involved in the pathogenesis of both acquired and familial type HLH.

**Method:**

The ION TORRENT semi-conductor sequencing method was used to sequence samples from 10 patients who were diagnosed or highly suspected of HLH. Then SNP rs2303116 of STXBP2 genotyping was performed by Sanger sequencing method on samples from 24 patients with HLH and 182 normal controls. Genotype frequencies were then compared and tested by multivariate logistic regression. Finally, the potential impact of rs2303116 on splicing factor binding ability was evaluated using the ESEfinder 3.0 online tool.

**Results:**

A total of 92 variants were identified in 10 HLH patients, of which 24 variants were rare variants (MAF<0.01), while the remaining 68 variants were common variants (MAF>0.01). Among them, 8 different genetic variations in the STXBP2 sequence were identified. We focused on the synonymous SNP rs2303116, as 30% of patients had CT/TT genotype. SNP genotyping was further performed on 24 HLH patients and 182 healthy control cohorts, and the results indicated a significantly elevated CT/TT genotype frequency of rs2303116 in HLH patients compared with healthy controls (patients 37.5% VS. controls 13.2%, P = 0.009, OR = 3.900, 95% CI 1.537–9.899). Multivariate logistic regression analysis indicated that being female (OR 0.350, 95% CI 0.143–0.861, P = 0.018) and of an older age (>43y, OR 0.312, 95% CI 0.118–0.822, P = 0.014) were independent protective factors, and the rs2303116 CT/TTgenotype (OR 3.900, 95% CI 1.537–9.899, P = 0.009) was an independent risk factor for HLH pathogenesis. By comparing the clinical parameters between HLH patients with CT/TT and CCgenotypes, we found that the patients with CT/TT genotype had significantly lower levels of fibrinogen, indicating more aggravated macrophage activation. In silico analysis of splice factor binding to rs2303116 CT/TT genotypes showed significant decrease for SRSF1 but increase for SRSF6, which suggested abnormal splicing machinery was associated with HLH pathogenesis.

**Conclusion:**

Our study demonstrated for the first time that HLH patients had significantly higher frequencies of the STXBP2 gene polymorphism rs2303116 variant compared with a healthy Chinese Han population, through clinical comparisons and further predictions we suggested regulation of alternative splicing by alleles of SNP rs2303116 could be involved in HLH pathogenesis.

## Introduction

Hemophagocytic lymphohistocytosis (HLH), also known as hemophagocytic syndrome (HPS), is an uncommon, life-threatening, hyperinflammatory immunological disorder that is characterized by prolonged high fever, pancytopenia, hyperlipidemia, hepatosplenomegaly and hemophagocytic phenomena in bone marrow slides [1]. As a hallmark of the disease, patients with HLH have notably impaired or diminished function of cytotoxic cells, including NK cells and cytotoxic T cells (CTLs) [2]. The impaired cytotoxicity not only reduces the ability of the immune system to eradicate invading pathogens, particularly viral infections, but also hampers its antigen-clearing functions, which allows it to accurately tailor the degree of the immune reaction. In turn, the uncontrolled proliferation and expansion of T cells and macrophages leads to so-called “cytokine storm”, which accounts for the syndrome severity and high mortality rate of HLH patients.

With the development of research and our understanding of the disease, it is well accepted that the two major subtypes of HLH can be classified based on the age of disease onset, family history, and the identification of a series of genetic variations that are responsible for the decreased functions of T/NK cells, such as PRF1/STXBP2/ITK and others. Generally, if patients have recurrent HLH manifestations during infancy or early childhood, with or without confirmation of positive family history, combined with the identification of germline mutations in HLH-related genes, “familial HLH” or “primary HLH” is suggested. On the other hand, “acquired HLH” or “secondary HLH” is considered if no obvious genetic aberrations are found and prior potential triggering events such as infection or malignancy are indicated [3].

Currently, the emergence of more evidence has blurred the boundary between primary and secondary HLH. Researchers have shown that multiple genetic factors can impact the cytotoxic pathway of T/NK cells by decreasing the threshold levels that result in uncontrolled immune cell activation after certain environmental triggers occur [4][5][6]. Therefore, the exact role that genetic aberrations play in the pathological process of HLH still remains to be demonstrated.

In this study, by utilizing the advanced technology of next-generation sequencing, we aimed to identify the potential genetic factors that play roles in the pathogenesis of or predisposition to HLH. Furthermore, we demonstrated the potential biological effects of the genetic variations that might participate in the occurrence and aggravation of HLH symptoms.

## Methods and Materials

### Sample collection and preparation

We retrospectively searched clinical data from diagnosed or highly suspected HLH patients who were admitted to the Department of Hematology, Tongji Hospital, Wuhan, Hubei, China between August 2010 and October 2014. Diagnosis was based on the major five criteria of the HLH-2004 protocol [7]. All patients with HLH (met the 5 criteria in the HLH-2004 protocol) as well as those highly suspected of HLH (met 4 of 5 criteria in the HLH-2004 protocol) were included in this study. These patients all went through a series of laboratory tests during initial diagnostic process, and the detailed type of these tests and corresponding normal range were listed in S1 Table. All necessary clinical data, such as blood test, liver function, and coagulation test results, were collected and analyzed, while 182 blood samples from ethnically matched healthy controls were collected from individuals who came for routine health examination from May 2014 to October 2014. The study was approved by the Ethics Review Board of Wuhan Tongji Hospital. All the studies involving human subjects were conducted in full compliance with government policies and the Declaration of Helsinki. Their written-form informed consents were obtained, and that the author's institutional review board has approved the study.

Bone marrow samples from the patients and whole blood from the healthy controls were collected and mononuclear cell isolation was performed by Ficoll density gradient centrifugation. A QIAamp DNA Blood Mini Kit (Qiagen, Hilden, Germany) was used for the preparation of genomic DNA. Total RNA was extracted using TRIzol reagent (Invitrogen) and reverse transcription (RT) was done employing a RevertAid first strand cDNA synthesis kit (Fermentas).

### Ion Torrent Next-Generation Sequencing

#### Primer Design

The design of primers targeted for 10 HLH-related genes was performed with the Ion AmpliSeq^™^ Ready-to-Use custom designer platform following the guide from the website (https://www.ampliseq.com/protected/dashboard.action). Three primer pools were mixed and provided by Life Technologies (Carlsbad, California, USA) to maximize the ability to perform ultrahigh-multiplex PCR reactions in one tube in parallel. Ultimately, 99.38% of the 43.66-kb targeted gene region was overlapped by 348 amplicons. Details for the primers used for genetic variation screening of HLH-related genes are available in S2 Table.

#### Library building

Ion Torrent libraries were built using the Ion AmpliSeq^TM^ Library Kit 2.0 (Life Technologies) following the manufacturer’s protocol. In general, 20 ng of gDNA per sample was used for multiplex PCR amplification, divided into 3 independent primer pools. Then, the products of the initial PCR amplifications were mixed together, partially digested, and ligated to barcodes and Ion Torrent adapters (Life Technologies). Afterwards, library purification was performed using AMPure XP beads (Beckman Coulter, Brea, CA, USA). Finally, the libraries were quantitated with a Qubit 2.0 fluorometer.

#### Emulsion PCR/Enrichment/Sequencing

After proper quantification of the libraries built for each sample, 10 patients were pooled together equally for application to an Ion Torrent 316 chip. The Ion Torrent OneTouch system (Life Technologies) and ES system (Life Technologies) were utilized for emulsion PCR and enrichment of the sequencing beads of the pooled libraries based on the instructions in the manufacturer’s protocol. Finally, an Ion Torrent Personal Genome Machine (PGM) (Life Technologies) and Ion Torrent 316 chip were used for HLH target gene sequencing. Raw data processing and VCF file generation were performed locally using the Ion Torrent platform-specific software Torrent Suite **v3.4.2.** The Ion Torrent online annotation platform Ion Reporter **V4.4** was used for further detailed analysis of genetic variants.

### STXBP2 SNP validation

A primer pair (forward: 5’-GGCTTCAGGGACCAGGGACG-3’/reverse: 5’–GGGATGGGGTCAAGGATGGGT-3’) was designed for STXBP2 SNP rs2303116 validation. The 406-bp PCR product was subjected to direct Sanger sequencing to detect variants.

### Bioinformatic analysis

To explore the potential effects of the synonymous SNP detected in this study, an online software was used (ESEfinder 3.0, rulai.cshl.edu/cgi-bin/tools/ESE3/esefinder.cgi?process = home) to predict the location of the exonic splice enhancer motif. Both the CC and CT/TT genotype 40-bp sequence were analyzed for possible binding sites for splice enhancers, including SRSF1/SRSF2/SRSF4/SRSF6.

### cDNA sequencing

To detect the potential events of STXBP2 mRNA splicing, cDNA obtained from HLH patients’ blood samples were subject to Sanger sequencing on exon7. Primer used in cDNA sequencing is Forward: 5’-CGCATCTTGTCTTCCTGCTG-3’/Reverse: 5’–ACCTGTATGTGTCCTGCTCT-3’; Polymerase chain reaction (PCR) was performed and the PCR product was subjected to direct Sanger sequencing.

### Statistical analysis

All major statistical analyses were performed using SPSS 21.0 (International Business Machine Corp., Armonk, NY, USA). All *P* values were two-sided, and the significance level was at least P<0.05. All patients and healthy controls were divided into two age groups (young and old) based on median age value. Differences in the frequencies for the age groups and sex between HLH patients and controls were compared with the *Χ*^*2*^ test. A Hardy-Weinberg equilibrium deviation test was performed using an online calculator tool [8] (http://www.oege.org/software/hwe-mr-calc.shtml), and the Rs2303116 genotypes of patients and controls were categorized into two groups(CT/TT genotype group, CC genotype group), and the genotype frequency distribution was first compared by Fisher’s exact test, and then binary logistic regression analysis was employed to investigate the potential association between STXBP2 SNP rs2303116 and the relative risk of HLH occurrence, in which age and sex were considered as covariates. Therefore, odds ratios (ORs) and 95% confidence intervals (95% CI) were calculated.

## Results

### Variant spectrum in HLH patients

To investigate the potential impact of genetic variants in HLH occurrence, we performed Ion Torrent sequencing analysis of 10 DNA samples extracted from patients admitted to our hospital who were diagnosed with HLH (met 5 criteria in the HLH-2004 protocol) or highly suspected of HLH (met 4 of 5 criteria in the HLH-2004 protocol).

The characteristics of patients recruited in this study are summarized in Table 1.

**Table 1:** Characteristic of HLH patients included in this research.

Patient characteristics		
**Age (y)**		
	Median	18
	Range	3–66
**Sex n/N (%)**		
	Male	16/24 (66.7)
	Female	8/24 (33.3)
**Diagnostic Criteria of HLH: n/N (%)**		
	Fever	20/24 (83.3)
	Hepatosplenomegaly	16/24 (66.7)
	Cytopenias	19/24 (79.2)
	Hypertriglyceridemia /hypofibrinogenemia	15/24 (62.5)
	Hemophagocytosis in bone marrow	12/24(50)
	Hyperferritinemia	20/24 (90)
**Related Clinical Menifestation**		
	EBV infection	6/24(25)
	Malignancy	8/24(33.3)

The panel designed for HLH sequencing analysis contained 10 genes, the variations of which are commonly detected in patients with familial type HLH (FHL), and several genetic diseases predisposing to HLH. These genes include PRF1 (related with FHL type 2), UNC13D (related with FHL type 3), STXBP2 (related with FHL type 5), STX11 (related with FHL type 4), SH2D1A (related with X-linked proliferative syndrome type 1, XLP), XIAP (related with X-linked proliferative syndrome type 2, XLP2), RAB27A(related with Griscelli Syndrome 2), LYST(related with Chediak–Higashi Syndrom), AP3B1(related with Hermansky–Pudlak

Syndrome), and ITK(related with ITK deficiency)[7]. The total number of amplicons for the panel was 348, with a total of 43,392 bases in the target region. Regarding the quality of the chip run, the median read length for the loaded samples was 160 bp. Overall, the total amount of base data reached 510 M. For each sample loaded, the average base coverage was 856.25, with an average of 321,419 reads mapped per sample and an average of 95.5% uniformity.

Overall, we identified a total of 92 DNA variants in this study, of which 24 variants were rare variants (MAF<0.01), including 2 synonymous, 7 non-synonymous, and 15 non-coding variants. The remaining 68 variants were common variants (MAF>0.01), including 14 synonymous, 3 non-synonymous, and 51 non-coding variants (detailed summary of all variants with MAF>0.01 was listed in S3 Table). A detailed summary of genetic variations is listed in Table 2.

**Table 2:** Ion Torrent Sequencing summary of patients diagnosed or highly suspected to have HLH (n = 10).

Sequencing Summary		
**No. of genes tested**		10
**No. of exons**		166
**Genomic sequence covered, bp**		43392
**No. of total variants**		92
**No. of rare variants (MAF< 1%)**		
	Total	24
	Synonymous	2
	Non-Synonymous	7
	Non-coding	15
**No. of common variants (MAF>1%)**		
	Total	68
	Synonymous	14
	Non-Synonymous	3
	Non-coding	51
**No. of novel SNPs covered**		8
**No. of known dbSNPs covered**		84

In this study, we concentrated our focus on the genetic variations specific to STXBP2, with variants comprising up to 1/3 of all rare variants. There were a total of 8 different genetic variations in the STXBP2 sequence, of which 4 were exonic variants (Table 3). Flanking 10 bp sequence of all rare variants of STXBP2 were listed in S4 Table.

**Table 3:** STXBP2 variation summary.

Gene	Region	Variant Type	Location	Exon	dbSNP*	Protein Change	MAF	Mutation Count n/N (%)
**STXBP2**	intronic	-	chr19:7704740	-	rs113656550	-	0	1/10 (10%)
**STXBP2**	exonic	synonymous	chr19:7705592	5	-	-	0	1/10 (10%)
**STXBP2**	exonic	synonymous	chr19:7706656	7	rs2303116	-	0.014	3/10 (30%)
**STXBP2**	splicesite_5	-	chr19:7707087	-	-	-	0	2/10 (20%)
**STXBP2**	exonic	missense	chr19:7707090	9	-	p.Gly233Ala	0	1/10 (10%)
**STXBP2**	exonic	frameshiftDeletion	chr19:7707327	10	rs187320742	p.Leu282fs	0.001	2/10 (20%)
**STXBP2**	intronic	-	chr19:7707758	-	rs117129319	-	0.008	1/10 (10%)
**STXBP2**	intronic	-	chr19:7710054	-	-		0	1/10 (10%)

* dbSNP build 142.

Specifically, the variant site at chr19:7706656 was included in our analysis because of its “borderline” MAF value (0.014) and high variant frequency (30%). Further variant annotations by Ion Reporter software indicated that chr19:7706656 variant STXBP2 c.528C>T was actually a SNP (rs2303116). Obvious differences in rs2303116 variant frequencies existed between the HLH patients tested in our study and sampled cohorts tested in the Hapmap and dbSNP databases(Hapmap database rs2303116 MAF value 0.048/dbSNP EAS population MAF value 0.056), and there is a possible role for the SNP in patient susceptibility to the disease. We further performed SNP validation of all available samples from HLH patients admitted in our hospital and a healthy control cohort.

### SNP screening in the HLH and healthy control cohorts

A total number of 24 patients who were diagnosed with or highly suspected of HLH and 182 ethnically matched healthy controls who were admitted for routine health examination were recruited in our study. Characteristics of the subjects are listed and statistically compared in Table 4.

**Table 4:** Univariate and multivariate analysis of patients and ethnically matched control populations in SNP genotyping study.

	Patients	Controls	Univariate			Multivariate^※^		
	N(%)	N(%)	OR	95%CI	P	OR	95% CI	P
**Age Group**^1^			0.312	0.118~0.822	0.014*	0.248	0.087~0.704	0.009
≤43	18(75)	88(48.3)						
>43	6(25)	94(51.6)						
**Sex**			0.350	0.143~0.861	0.018*	0.352	0.137~0.905	0.030
Male	16(66.7)	75(41.2)						
Female	8(33.3)	107(58.8)						
**rs2303116**			3.900	1.537~9.899	0.009^¶^	5.116	1.847~14.173	0.002
CC	15(62.5)	156(85.7)						
CT or TT	9(37.5)	24(13.2)						
Unknown	0(0)	2(1.1)						

^1^. Age group was divided by the median number (43y) of age from patients and healthy cohorts

^¶^P value was calculated by Fisher’s Exact Test

*P value was calculated by Pearson Chi-Square test

^※^P value was calculated by Logistic Regression analysis.

The median age of HLH patients was 18 years, ranging from 3 to 66 years, which was significantly lower than the healthy cohort, which had a median age of 50 years, ranging from 16 to 88 years (P<0.05); based on this, we determined the young (≤ 43y) and old (>43y) age groups, and significant differences existed in terms of composition between HLH patients and healthy cohorts. In terms of gender composition, we found that the percentage of male patients was significantly higher compared with the healthy cohort (66.7% VS. 41.2%, P = 0.018).

**Fig 1:**
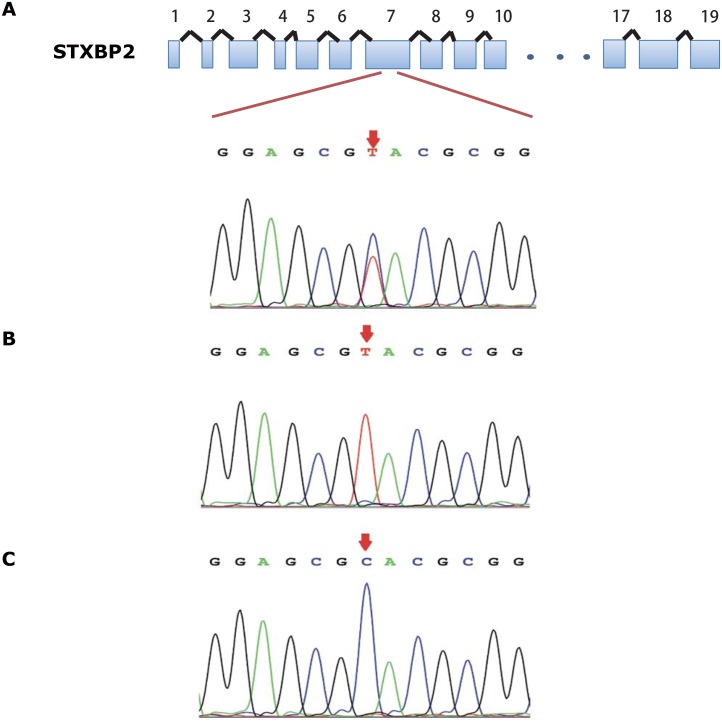
Detection of STXBP2 c.528C>T heterozygous /homozygous SNPs in patients’ samples. Chromatograms of STXBP2 coding region sequences from (A)CT genotype, (B)TT genotype, and (C) CC genotype patients’ samples were shown.

We then performed SNP rs2303116 genotyping using the Sanger sequencing method for all samples obtained. A total of 24 HLH patient samples and 180 healthy control samples were successfully sequenced, while 2 control samples failed in sequencing due to low DNA quality. Typical chromatograms of CC, CT and TT genotypes sequences are shown in Fig 1. In the control cohort, no significant deviations from Hardy-Weinberg equilibrium were identified (χ^2^ = 0.92, P>0.05). We found significant differences in genotype distribution between HLH patients and healthy controls, with the genotype CT and TT frequency significantly elevated in comparison to the healthy cohort (patients 37.5% VS. controls 13.2%, P = 0.009, OR = 3.900, 95% CI 1.537–9.899). To minimize the impact of covariates such as age and sex, binary logistic regression analysis was performed and the significance of the genotype differences was retained (P = 0.002, OR = 5.116, 95% CI 1.847–14.173). In summary, the results indicated that HLH patients, regardless of etiology, had significantly higher CT and TT genotype frequencies of STXBP2 SNP rs2303116 compared with the normal control cohort.

### Differences in clinical characteristics between rs2303116 CT/TT genotype and CC genotype patients

To investigate the possible influences of the variant on disease occurrence and the pathological process, we collected relevant clinical information from all patients included in the SNP genotyping study. The clinical parameters from the initial diagnosis of patients with CT/TT genotype and CC genotype were compared and listed in detail (Table 5).

**Table 5:** Difference in clinical characteristics of HLH patients with STXBP2 rs2303116 CT/TT genotypes and CC genotype.

Clinical Characteristics (Mean value and range)	CT/TT genotype patients	CC genotype patients	P value*
PLT (*10^**9**^**/L)**	41.74 (5.2~133)	36.74 (11~113)	0.952
WBC (*10^**9**^**/L)**	2.46 (0.01~5.85)	2.11 (0.08~6.52)	0.676
**Fibrinogen (g/L)**	0.97 (0.5~1.73)	1.78 (0.5~4.24)	**0.036**
**Ferritin (μg/L)**	45350.80 (1130~168620)	18619.57 (453~134000)	0.241
**Albumin (g/L)**	31.08 (24~40.7)	29.33 (19.9~41.4)	0.511
**ALT (U/L)**	424.11 (15~2583)	163.27 (24~416)	1.000
**AST (U/L)**	313.78 (26~1712)	88.33 (14~268)	0.456

* P values were calculated by Mann-Whitney test.

Unfortunately, we failed to identify significant differences in levels from blood tests (PLT, WBC) or liver function (albumin, ALT, AST). However, there was an elevation trend for ferritin levels in CT/TT genotype patients (45350.80 μg/L VS. 18619.57 μg/L), although it was not statistically significant (*P* = 0.241). In addition, we observed significantly decreased plasminogen levels (0.97 g/L VS. 1.78 g/L, P = 0.036) in the CT/TT genotype patient group. As ferritin and plasminogen levels both indicate the degree of macrophage activation, our results indicated that much more aggravated macrophage activation might occur in CT/TT genotype patients compared with their wildtype counterparts. Furthermore, the results further suggested that patients with CT/TT genotype might have much more impaired cytotoxic cell functions, such as those of NK cells and cytotoxic T cells, due to the functional abnormalities of granule exocytosis conferred by STXBP2 and its interaction with STX11. However, the detailed mechanisms underlying the STXBP2 SNP and its hampered gene function require further clarification.

### In silico analysis of rs2303116 impacts the STXBP2 splicing enhancer binding motif

**Fig 2:**
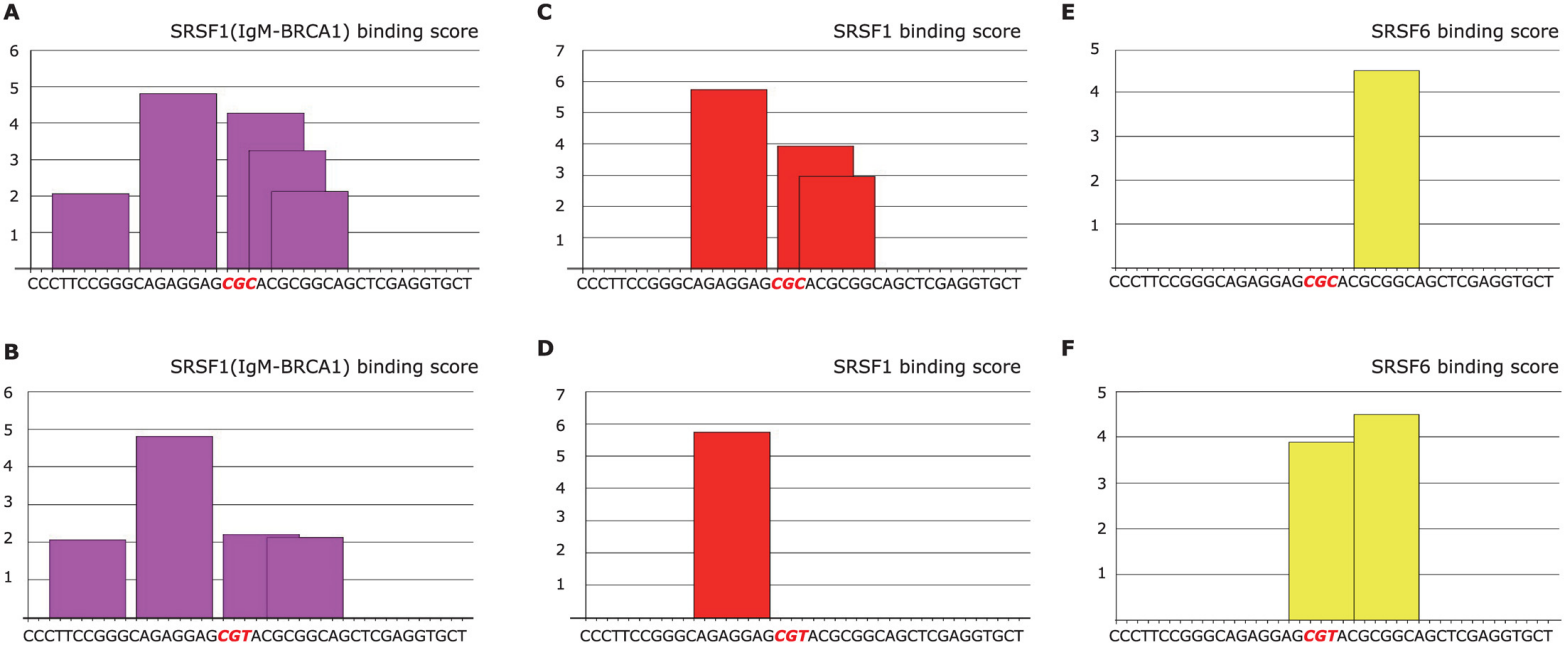
*In silico* analysis of potential disruption of STXBP2 variation on exonic splicing elements (ESEs), by using online analytical tool ESEfinder (http://rulai.cshl.edu/cgi-bin/tools/ESE3/esefinder.cgi?process=home). Binding score predictions for SRSF1/SRSF1(IgM-BRCA1)/SRSF6 were listed separately. X-axis demonstrated sequences about 40 bps around STXBP2 variant site, Y-axis demonstrated prediction binding score of each splicing factor. (A) Predicted site and relative score of splicing enhancer binding motif for SRSF1 (IgM-BRCA1) in STXBP2 rs2303116 CC genotype sequence (40bp); (B) Predicted site and relative score of splicing enhancer binding motif for SRSF1 (IgM-BRCA1) in STXBP2 rs2303116 CT/TT genotype sequence (40bp); (C) Predicted site and relative score of splicing enhancer binding motif for SRSF1 in STXBP2 rs2303116 CC genotype sequence (40bp); (D) Predicted site and relative score of splicing enhancer binding motif for SRSF1 in STXBP2 rs2303116 CT/TT genotype sequence (40bp).

In our effort to further explain the connection between STXBP2 SNP rs2303116 with the clinical phenotype of patients, we evaluated the possible impact of the SNP on the binding of splicing enhancers such as SRSF1/SRSF3. An online prediction software platform, ESEfinder 3.0 (https://rulai.cshl.edu/cgi-bin/tools/ESE3/esefinder.cgi?process=home), was used to evaluate potential binding sites. First, we input 1000 bp of both the STXBP2 CT/TT genotype and CC genotype sequences (500 bp flanking each side of the SNP rs2303116 location) to evaluate the best-hit for splicing enhancer binding. A detailed description of the prediction procedure can been found in S1 File. As a result, we identified a SRSF1 family binding site with the highest prediction score (5.73874) just 10 bp upstream of the variant location (chromosome 19, STXBP exon 7), which prompted us to perform further explorations by investigating sequences proximal to the variant site. Forty one base pairs from both the STXBP2 CT/TT genotype and CC genotype sequences (20 bp flanking each side of the SNP rs2303116 location) were uploaded to predict SRSF1/2/5/6 binding sites. As is shown in Fig 2, we identified significantly reduced levels of SRSF1/SRSF1(IgM-BRCA1) binding within 9 bp of the variant site, while the binding affinity for SRSF6 significantly increased. The results indicated that rs2303116 might play a role in the occurrence of STXBP2 abnormal splicing and thus contribute to the pathological process of the disease. To confirm our hypothesis, we performed cDNA sequencing on RNA samples from CT genotype patients. Unfortunately, due to the very limited number of RNA samples that were available in our hospital, we failed to identify positive abnormal splicing events (cDNA sequencing result from one HLH patient with CT genotype was shown as an example in S2 File). Larger cohort experiments are still needed to confirm our analysis.

## Discussion

In this study, we utilized the high-resolution sequencing platform Ion Torrent PGM for variant detection in 10 HLH-related genes. NGS technology has been used for over a decade and has proven to be very efficient and economical for high-throughput sequencing tasks in comparison with traditional Sanger methods [9]. Given the heterogeneous nature of the disease and widely scattered distribution of variant sites in the gene sequences [10], massive parallel sequencing techniques could produce satisfactory results in an accurate and time/cost efficient manner [11][12][13].

With accumulating knowledge and experimental evidence gained from clinical studies and animal models, the distinctions between primary HLH and secondary HLH have been blurred. Studies have demonstrated that HLH is actually a singular syndrome with a continuum of risk factors [14]. Normally, lymphocyte cytotoxic function prevents the over-reactivation of T/NK cells, which results in a "high" threshold of HLH development. However, with the existence of variants influencing the cytotoxic immunity pathway, such a threshold would be lowered, and patients would develop HLH symptoms under strong immunological stimuli such as infections or autoimmune disease. In other words, primary HLH patients are actually those that bear severe genetic aberrations, such that minimal infections can trigger massive T cell reactions. Meanwhile, less devastating/hypomorphic aberrations, such as missense/splice site variants, are also likely to exist in secondary HLH patients. Given that these genes still retain residual function, only a limited number of immune stimuli can trigger the disease, such as Epstein-Barr viral infection or lymphoma [4]. Our study did support this theory by demonstrating that the frequency of STXBP2 SNP rs2303116, a type of common single nucleotide variation, was significantly increased in HLH patients, which indicated that gene polymorphisms might be involved in HLH susceptibility. Additionally, these results also suggested that the SNP alone was not sufficient to explain the occurrence of HLH in those patients; other factors such as prior immune stimulation or additional genetic aberrations should also be taken into consideration. However, due to the small number of our patient cohort, our research was of limited informative value as a genetic association study, and a much larger cohort collected in a multi-center collaboration would still be needed for further consolidation of our hypothesis.

**Fig 3:**
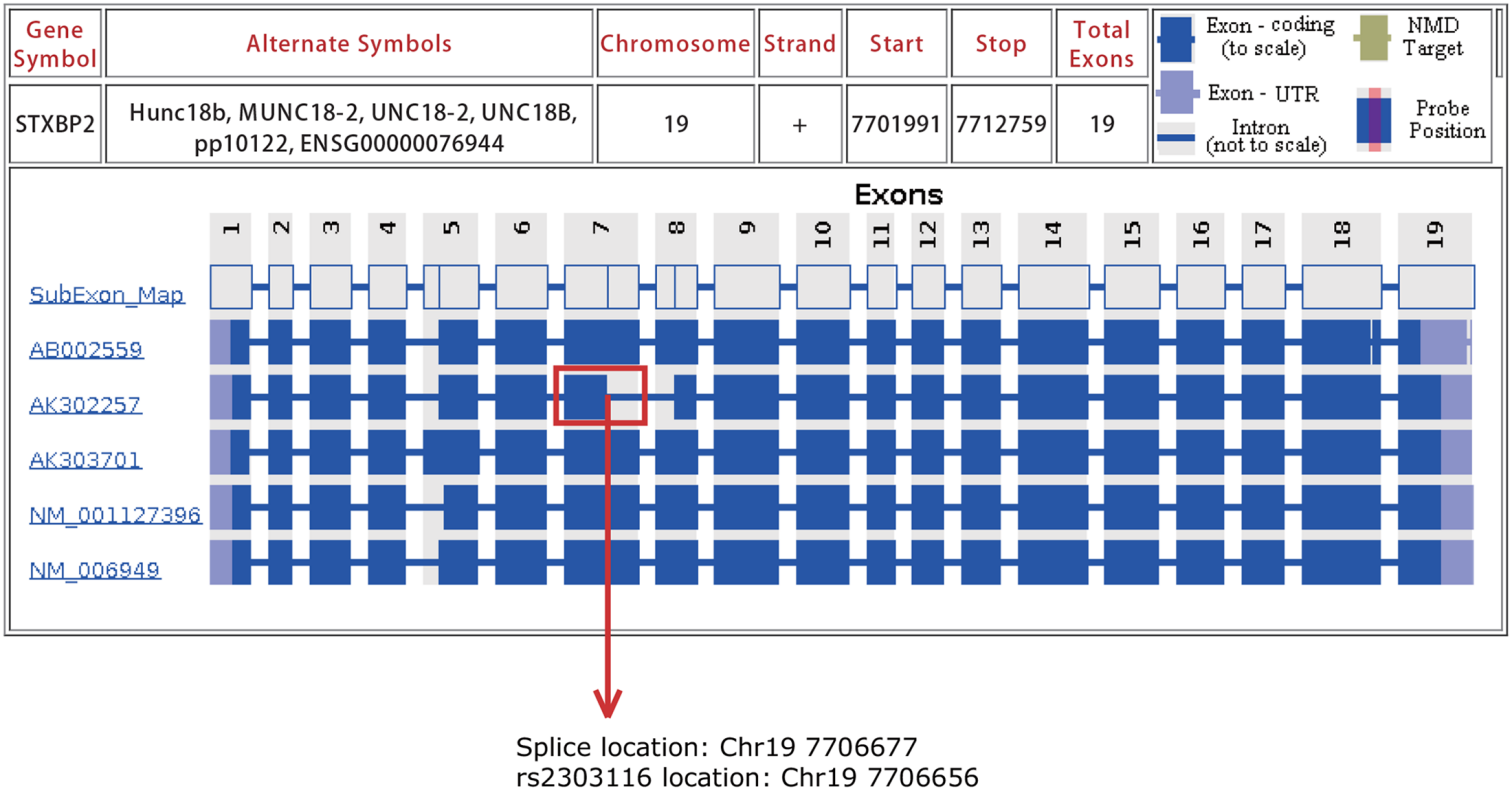
Database query for splice events of STXBP2 gene. By using online alternative splicing tool SpliceMiner(http://projects.insilico.us/SpliceMiner/Search), Exon7 splicing event was detected and emphasized by red rectangle.

Previous studies have identified a STXBP2 gene variant that is responsible for the pathogenesis of a HLH subtype, familial HLH type 5. Specifically, STXBP2 is a member of the Sec/Munc proteins, which are vital for SNARE complex assembly and disassembly as well as the control of membrane fusion. This gene plays an important role not only in platelet secretion [15] but also in the cytotoxic granule exocytosis of NK/T cells [16]. Therefore, decreased levels of STXBP2 expression and abnormal structure of the protein diminish the pathogen eradicating abilities of those cells [16][17][18]. A large cohort study of 37 FHL5 patients demonstrated that STXBP2 variants were scattered throughout the gene, and different types of variants were identified. FHL5 patients who carry missense variants had a higher risk of early onset, while those carrying splice site variants tended to have late onset and frequent mild reactivation [18]. Through *in silico* analysis of STXBP2 gene polymorphisms and splicing factor binding, it was suggested that the synonymous variant rs2303116 potentially impacted the splicing events for STXBP2 exon7. Unfortunately, we failed to identify alternatively-spliced STXBP2 sequence from our limited cDNA samples from CT genotype HLH patients. To explain such phenomenon, we believed the “dosage effect” of the variant might play a role in the splicing mechanism, which means the HLH patients with TT genotype might be more likely to had exon splicing than CT genotype. Due to the lack of samples from TT genotype patients, we were not able to perform cDNA sequencing on these patients; however, using the online splicing database SpliceMiner [19] (http://projects.insilico.us/SpliceMiner/Gene.jsp), we did find evidence of STXBP2 exon7 splicing events just 21 bp downstream of the variant site, which is shown in detail in Fig 3. In our study, we also observed significantly decreased levels of fibrinogen in STXBP2 CT/TT genotype patients compared with the CC genotype counterparts, which was indicative of more aggravated macrophage activation. However, how this alternatively-spliced of the STXBP2 protein affects its normal function and its association with HLH pathogenesis remain to be elucidated.

**Fig 4:**
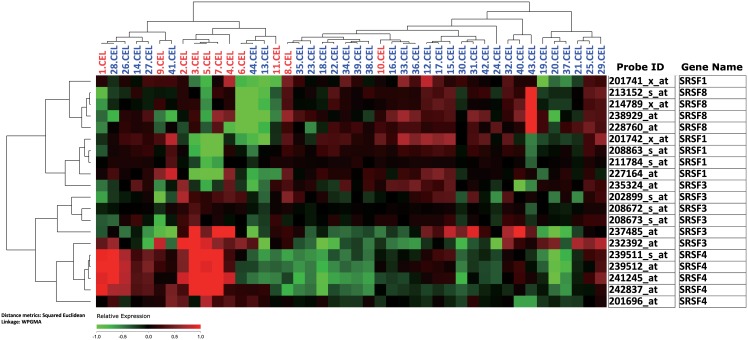
Unsupervised hierarchical cluster analysis of HLH patients and healthy cohort based on SRSF family expression pattern. Microarray analysis was performed using microarray analytical software package J-Express (Version 2012, developed by Department of Informatics, University of Bergen, Norway). 11 HLH patients and 33 healthy controls from GEO database (GSE26050) were clustered based on similarities of SRSF family genes which were differentially expressed. The cluster diagram of gene expression values was mean-centered. Samples of HLH patients were labeled with red ID marker ranging from 1.CEL to 11.CEL, while healthy controls were labeled with green ID marker ranging from 12.CEL to 44.CEL.

To further explore the role of SRSF families in the pathogenesis of HLH, we retrieved online microarray raw data from mononuclear cells from 11 HLH patients and 33 healthy controls in the GEO database (http://www.ncbi.nlm.nih.gov/geo/, accession number GSE26050). Detailed processing methods can be found in previous publications [20]. By comparing SRSF family expression levels between HLH patients and healthy controls, we identified slight but significant changes in SRSF family expression patterns between two groups, which are shown in Fig 4 and significantly differentiated expressed gene probes were listed in Table 6. There was a slight decrease in SRSF1 and increase in SRSF3/4 expression. Additionally,by dividing HLH patients into 2 groups according to the average expression value of SRSF1, we surprisingly found the HLH patients group with low SRSF1 expression level had significantly elevated STXBP2 expression level (unpaired t test, p = 0.048)(S1 Fig), such connections could be a factor that, when combined with gene polymorphisms, would modulate STXBP2 gene function and further influence the pathological process in HLH patients. However, such a hypothesis requires further experimental validation.

**Table 6:** Statistically significant expression level change of SRSF family genes in HLH patients identified by microarray analysis. Data were extracted from public database GEO (http://www.ncbi.nlm.nih.gov/geo, dataset accession number GSE26050), in which global gene expression pattern of mononuclear cells from 11 active HLH patients were compared with 33 normal controls. P values were calculated by unpaired student t test.

Probe ID	Gene Symbol	Patients Average*	Control Average*	Fold change	P Value
**201742_x_at**	SRSF1	7.884	8.456	0.673	0.042
**208863_s_at**	SRSF1	11.308	11.682	0.772	0.051
**208672_s_at**	SRSF3	12.387	12.302	1.061	0.028
**232392_at**	SRSF3	9.369	8.603	1.701	0.000
**201696_at**	SRSF4	7.995	7.765	1.173	0.010
**239511_s_at**	SRSF4	6.698	6.175	1.437	0.004
**241245_at**	SRSF4	7.927	7.288	1.557	0.002
**242837_at**	SRSF4	9.357	8.841	1.429	0.027

* Probe average values from patients and control groups are all in log2 form

## Conclusion

In our study, by utilizing modern Ion Torrent semi-conductor sequencing technology, we unexpectedly identified a common STXBP2 gene polymorphism rs2303116, which had significantly elevated frequencies in HLH patients compared with healthy controls from the Chinese Han population. Our study proposed that genetic susceptibility might be involved in the pathogenesis of HLH. By comparing clinical data, we found that patients with CT/TT genotypes had significantly decreased levels of fibrinogen, indicating that STXBP2 variant might impair protein function. Through *in silico* analysis, we identified the association of the SNP with potential abnormal splicing events in STXBP2 exon7. Further microarray analysis indicated a slight but significant change in SRSF family expression patterns, which implicated the SRSF family as well as splicing machinery in the pathogenesis of and susceptibility to HLH.

## Supporting Information

S1 Fig Comparison of STXBP2 expression value between SRSF1 high and SRSF1 low expression group in HLH patients. STXBP2 expression levels (probe 209367_at) were compared between 2 groups of HLH patients, namely SRSF1 high group and SRSF1 low group. These two groups were divided by the group average value of SRSF1 expression levels (average log2 value 8.986, combining probe 201741_x_at/201742_x_at/ 208863_s_at/211784_s_at/227164_at) from all 11 HLH patients. P value was calculated using unpaired t test.
(EPS)

S1 File ESEfinder prediction of SRSF family binding site around STXBP2 variant rs2303116 location. 1000 bp of both the STXBP2 CT/TT genotype and CC genotype sequences (500 bp flanking each side of the SNP rs2303116 location) were evaluated for the best-hit for splicing enhancer binding, including SRSF1/SRSF2/SRSF5/SRSF6 binding sites.
(PDF)

S2 File Result of CT genotype HLH patient exon7 cDNA sequencing. To detect the potential events of STXBP2 mRNA splicing, cDNA obtained from HLH patients’ blood samples were subject to Sanger sequencing on exon7. Primer used in cDNA sequencing is Forward: 5’-CGCATCTTGTCTTCCTGCTG-3’/Reverse: 5’–ACCTGTATGTGTCCTGCTCT-3’.
(PDF)

S1 Table Clinical tests and normal range. All relavant clinical tests of HLH patients treated in our hospital and corresponding normal range of each test were listed.
(XLSX)

S2 Table Primers used for genetic variation screening of HLH-related genes. Ampliseq (version 2.2) platform was used to designed HLH panel primers, which consisted of 3 pools and detailed sequence of each primer was listed below.
(XLSX)

S3 Table List of all variants with MAF>0.01. All variants with MAF>0.01 were listed and the related annotative information such as location, functional change and dbSNP number were listed in detail.
(XLSX)

S4 Table 10bp Flanking sequence of STXBP2 rare variants. 10 bp flanking sequence of all rare variants of STXBP2 gene were listed below.
(XLSX)
