# Supplementary material for: Hemophagocytic Lymphohistocytosis in the Chinese Han Population May Be Associated with an STXBP2 Gene Polymorphism
Source: PLoS One. 2016 Aug 11;11(8):e0159454. doi: 10.1371/journal.pone.0159454 (PMC4981359; doi:10.1371/journal.pone.0159454)
Supplement: S1 File — 1000 bp of both the STXBP2 CT/TT genotype and CC genotype sequences (500 bp flanking each side of the SNP rs2303116 location) were evaluated for the best-hit for splicing enhancer binding, including SRSF1/SRSF2/SRSF5/SRSF6 binding sites. (PDF) [file pone.0159454.s002.pdf]

Supplementary method:

1. **Input Sequence:**

>gi|224589810:7706156-7707156 Homo sapiens chromosome 19, GRCh37.p13 Primary Assembly
TGGGGTGGGAGAGTCGCTTGAGCCCGGGAGGTCGAGGCTGCAGTGAGCTATGATT
GCACTGCTGCCCTCCAGCCTGGGCGACAGAGCGAGATCCTGTCTCAAAAACATACAT
AAAGTAAAATTTTAAAAAGGGGAGGTACCCACAGAGTCCAAGGAGCTCTTGCCTTGA
GTTCTATCCCACTAGGCCCTTGCAGGGGGCAGCGTAGAGCGCACCGCGGGGTTGTC
CAGCCAGCTTAAGGGACACGGGCTGGGGTATTTATCCACTGACTCCTGCAGGCAT
GGGTTTAGGGTTGACCTGGGCCTGCCTCCAATTCGGCAAAGCAGGCTTCAGGGACC
AGGGACGGCTCCCAGGAGGTGCAGGTGGCGGCAGCGGGAAGCGGGGCAGGTGTG
CACCTGCAGCGGCAACCCTGGTGCTTCTGTCCCCTCCTCGCCCAGGTGTTCTCCCTC
GATGCTCCCCACAGCACCTACAACCTCTACTGCCCCTTCCGGGCAGAGGAGCGC/T
ACGCGGCAGCTCGAGGTGCTGGCCCAGCAGATTGCCACGCTGTGCGCCACCCTGCA
GGAGTACCCGGCCATCCGCTACCGCAAGTGGGGACCCCACCCAGCCCCACCCCGAT
GCCGACCCCCCCTTAACCGCGTGCAACACCTAACCTTTAACCTCTCTTGTGACCCCAG
CCCCGGCCCTACCCTGGCCCCTGACTCTCACCTTCAAACCCATCCTTGACCCCATCCC
CTGATGATGTCCCCCGTGTCTGACCTCCCCGCCAGGGGCCCAGAGGACACAGCCCA
GTTGGCCCACGCCGTCCTGGCCAAGCTGAACGCCTTCAAGGCAGACACTCCCAGTC
TGGGCGAGGTGAGGGGGCGTGCTTGGGAGGTGAGGGGCAGCCCCAACCGGCTCA
GGGTCAGTGCCTCATTCCTGCCCTAAACCCCACCCCAGGGCCCAGAGAAAACCCGC
TCCCAGCTGCTGATAATGGACCGGGCAGCTGACCCCGTGTCCCCACTACT

* NOTE: 1001bp of STXBP2 sequence (500bp flanking each side of rs2303116 locus) were retrieved and input into software. Letter in red indicated STXBP2 rs2303116 SNP location.

2. Prediction of best binding location of splicing enhancer

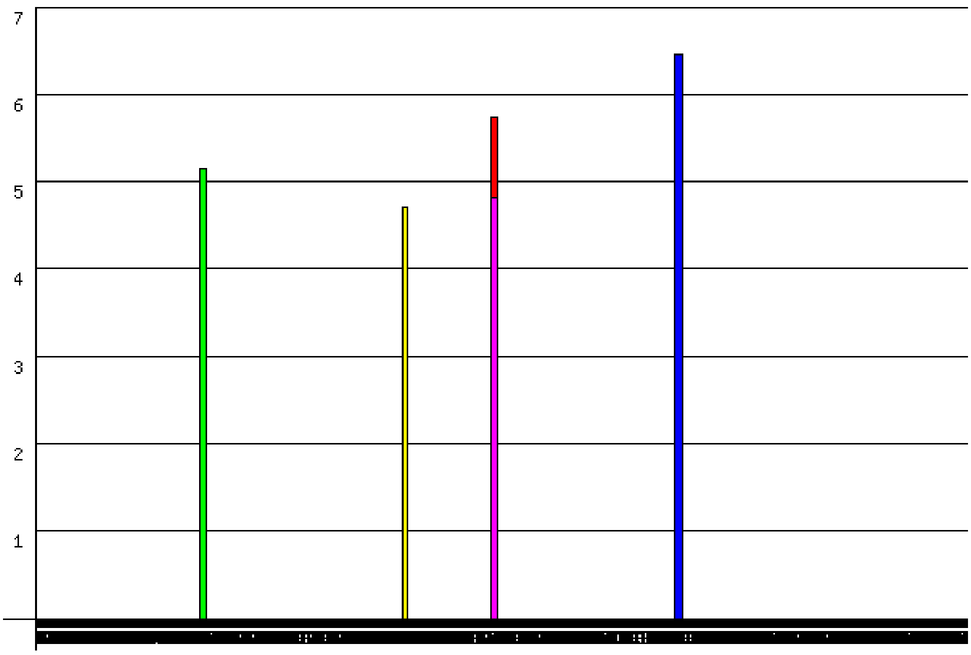

Figure: splicing enhancer locations with the highest prediction score were shown above.

Tabular results:

Detailed location and prediction score of splicing enhancer binding

SRSF1 threshold: 1.956	SRSF1 (IgM-BRCA1) threshold: 1.867	SRSF2 threshold: 2.383	SRSF5 threshold: 2.67	SRSF6 threshold: 2.676
Position*/Site/Score	Position*/Site/Score	Position*/Site/Score	Position*/Site/Score	Position*/Site/Score
491 (-511) CAGAGGA 5.73874	491 (-511) CAGAGGA 4.81171	687 (-315) GGCCCCTG 6.45166	178 (-824) CCACTAG 5.15415	395 (-607) TGCAGC 4.71335

* both positions from 5'end (through 1) and 3'end (through -1) are given
