# Supplementary figures and images for: Hemophagocytic Lymphohistocytosis in the Chinese Han Population May Be Associated with an STXBP2 Gene Polymorphism
Source: PLoS One. 2016 Aug 11;11(8):e0159454. doi: 10.1371/journal.pone.0159454 (PMC4981359; doi:10.1371/journal.pone.0159454)

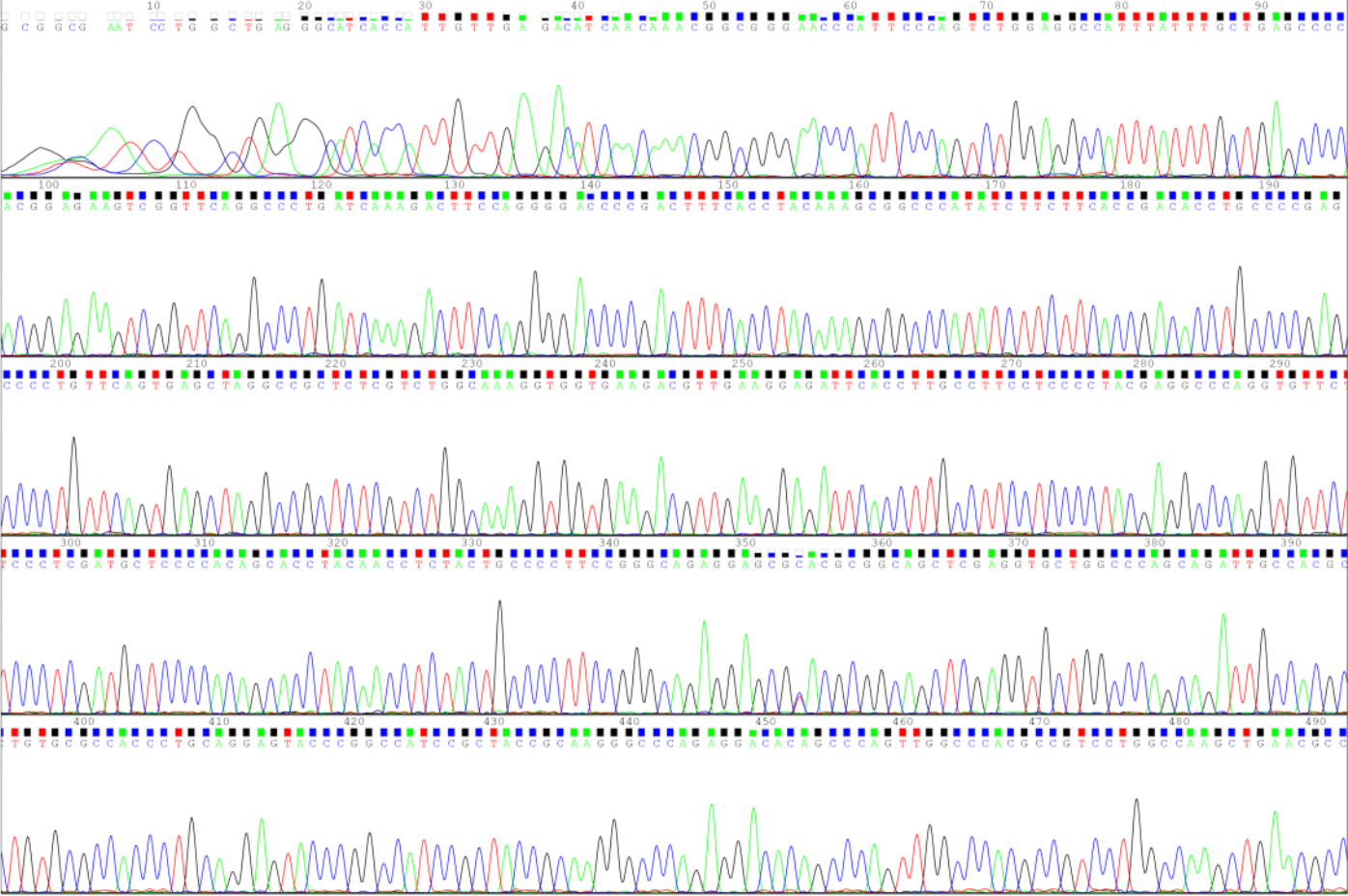

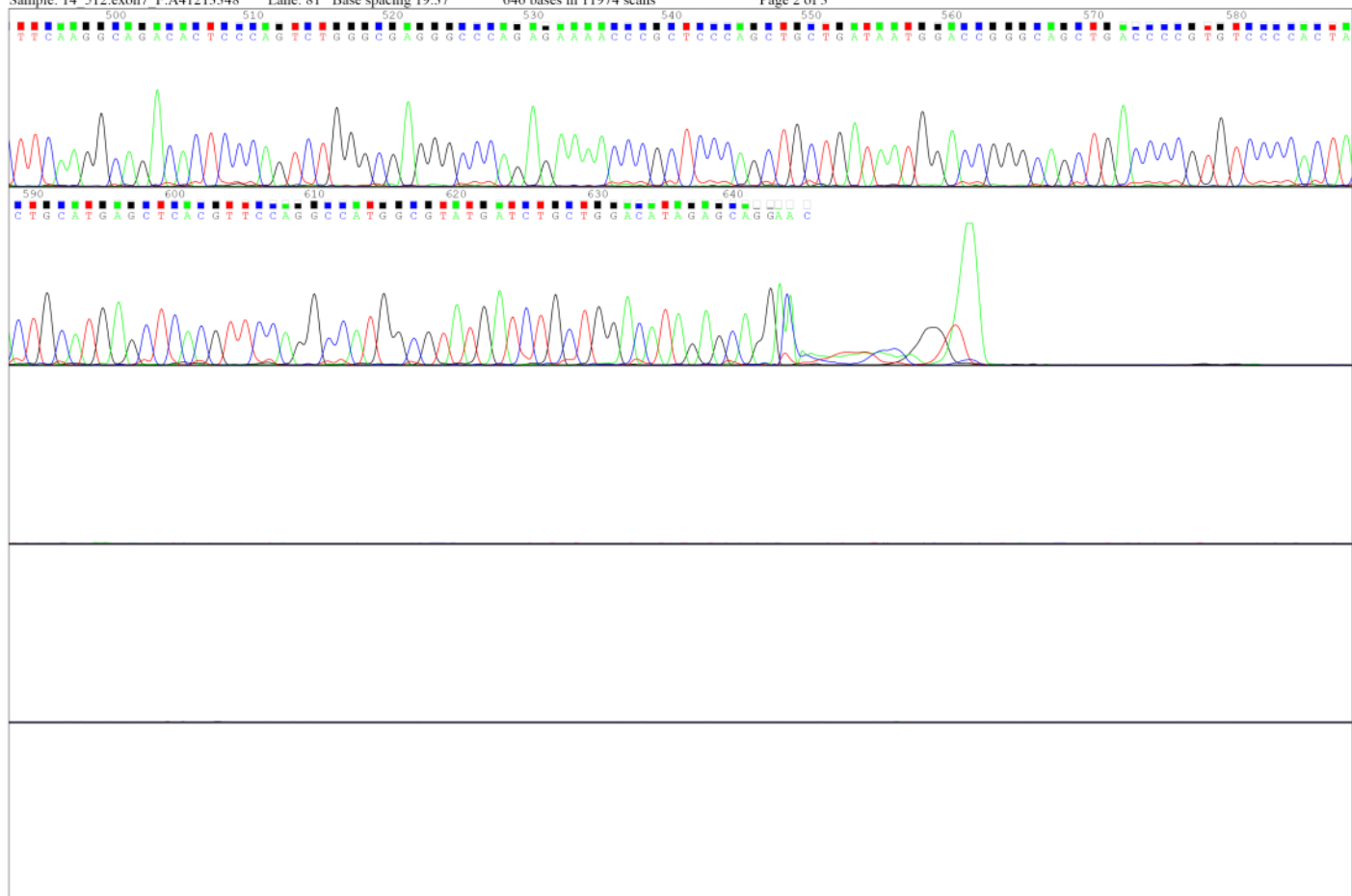

Supplement: S2 File — To detect the potential events of STXBP2 mRNA splicing, cDNA obtained from HLH patients’ blood samples were subject to Sanger sequencing on exon7. Primer used in cDNA sequencing is Forward: 5’-CGCATCTTGTCTTCCTGCTG-3’/Reverse: 5’–ACCTGTATGTGTCCTGCTCT-3’. (PDF) [file pone.0159454.s003.pdf]
